# Cross-species comparative analysis of Dicer proteins during Sindbis virus infection

**DOI:** 10.1038/srep10693

**Published:** 2015-05-29

**Authors:** Erika Girardi, Mathieu Lefèvre, Béatrice Chane-Woon-Ming, Simona Paro, Bill Claydon, Jean-Luc Imler, Carine Meignin, Sébastien Pfeffer

**Affiliations:** 1 Architecture and Reactivity of RNA, Institut de Biologie Moléculaire et Cellulaire du CNRS, Université de Strasbourg, 15 rue René Descartes, 67084 Strasbourg, France; 2 Immune response and development in insects, Institut de Biologie Moléculaire et Cellulaire du CNRS, Université de Strasbourg, 15 rue René Descartes, 67084 Strasbourg, France

## Abstract

In plants and invertebrates RNA silencing is a major defense mechanism against virus infections. The first event in RNA silencing is dicing of long double stranded RNAs into small interfering RNAs (siRNAs). The Dicer proteins involved in this process are phylogenetically conserved and have the same domain organization. Accordingly, the production of viral derived siRNAs has also been observed in the mouse, but only in restricted cell types. To gain insight on this restriction, we compare the dicing activity of human Dicer and fly Dicer-2 in the context of Sindbis virus (SINV) infection. Expression of human Dicer in flies inefficiently rescues the production of viral siRNAs but confers some protection against SINV. Conversely, expression of Dicer-2 in human cells allows the production of viral 21 nt small RNAs. However, this does not confer resistance to viral infection, but on the contrary results in stronger accumulation of viral RNA. We further show that Dicer-2 expression in human cells perturbs interferon (IFN) signaling pathways and antagonizes protein kinase R (PKR)-mediated antiviral immunity. Overall, our data suggest that a functional incompatibility between the Dicer and IFN pathways explains the predominance of the IFN response in mammalian somatic cells.

RNA interference (RNAi) plays an essential role in gene regulation and defense against viruses in most eukaryotes^1^. This defense pathway is triggered by the recognition, as a foreign element, of a double-stranded RNA (dsRNA) molecule^1^,^2^ and is followed by its processing into small interfering RNAs (siRNAs) by Dicer proteins. siRNAs are then loaded into an Argonaute-containing-RNA-induced silencing complex (RISC) and guide the complex towards target viral RNAs for silencing^3^. Dicer proteins represent a widely conserved family of RNase III endoribonucleases, which specifically and progressively cleave dsRNAs into smaller duplex fragments of discrete sizes *in vitro*, starting preferentially from the extremities^4^. From fungi to higher eukaryotes, Dicer proteins are essential for the biogenesis of both micro (mi)RNAs and siRNAs^5^,^6^. These multi-domain proteins contain a substrate recognition domain (PAZ domain), a divergent dsRNA-binding domain, two tandem RNase III domains and an additional dsRNA-binding domain^7^. Most of Dicer RNases also encode a N-terminal DExD/H RNA heli-case domain closely related to that of RIG-I like receptors (RLRs), which act as sensor for viral RNAs in the interferon (IFN)-mediated response to viruses in vertebrates^8^. The IFN pathway represents the major cellular response to viruses in mammals, and involves the induction of IFN-stimulated genes (ISGs) to block the viral replication and/or lead to programmed cell death^9^.

The number of Dicer proteins varies among organisms. Thus, mammalian genomes only encode one Dicer protein required for both siRNA and miRNA-silencing pathways^10^,^11^. In contrast, the *D. melanogaster* genome encodes two functionally distinct Dicers: Dicer-2 produces siRNAs from long dsRNA precursors, whereas Dicer-1 recognizes stem-loop structures present in miRNA precursors^12^.

Several proteins have been identified as partners of Dicer. For instance, the fly Dicer-1 interacts with the double-stranded RNA binding protein (dsRBP) Loqs-PB for miRNA biogenesis 13,14. Although the generation of endo-siRNAs by Dicer-2 requires another isoform of Loqs, Loqs-PD^15^, the production of exogenous and viral siRNAs requires the formation of an alternative complex between Dicer-2 and the protein R2D2^16^. Dicer-2 on its own is able to process dsRNAs, however the resulting siRNAs are not effectively loaded into the RISC complex^17^,^18^. It has also been proposed that interaction of Dicer-2 with R2D2 prevents its binding to miRNA precursors *in vitro*^19^. In human, the unique Dicer (hDicer) interacts with two dsRBP partners, TRBP (TAR RNA binding protein) and PACT (protein activator of the interferon-induced protein kinase), which facilitate the positioning and loading of miRNAs into RISC^20^. Both proteins have been previously identified as regulators of protein kinase R (PKR), a dsRNA-activated eIF2α kinase encoded by an ISG and involved in the shut-down of translation in virus-infected cells^21^,^22^.

It has been postulated that the evolution of the IFN system in vertebrates has supplanted RNAi as the major defense system against viral infection^23^. This hypothesis was recently put to the test with two reports providing evidence for antiviral RNAi in mouse embryonic stem cells (mESCs) and neonate animals^24^,^25^. However, it remains unclear whether RNAi is indeed a relevant component of innate antiviral immunity in differentiated mammalian cells^26^,^27^,^28^ and why the structurally similar proteins Dicer-2 and hDicer have different properties *in vivo*.

In order to address these questions, we established an experimental approach based on the use of Sindbis arbovirus (SINV), which is able to infect both mammalian and insect cells. We generated transgenic flies expressing tagged hDicer or Dicer-2 in *dicer-2* null mutant background. In parallel, we stably expressed *D. melanogaster* Dicer-2, alone or with its partner R2D2, in HEK293 cells. We then measured the impact of the heterologous protein expression on the production of virus-derived small RNAs (sRNA) and the antiviral response in the two host systems. Our results show that the stable expression of hDicer in *dicer-2*-deficient flies does not rescue RNAi but does display a measurable antiviral function. On the other hand, although the Dicer-2-R2D2 complex generates viral 21 nt small (s)RNAs in HEK293 cells early during infection, this does not seem sufficient to mediate antiviral effects. On the contrary, Dicer-2 expression seems to impair IFN signaling and disturbs PKR function.

## Results

### hDicer transgenic flies display a partial antiviral function

**Figure 1:**
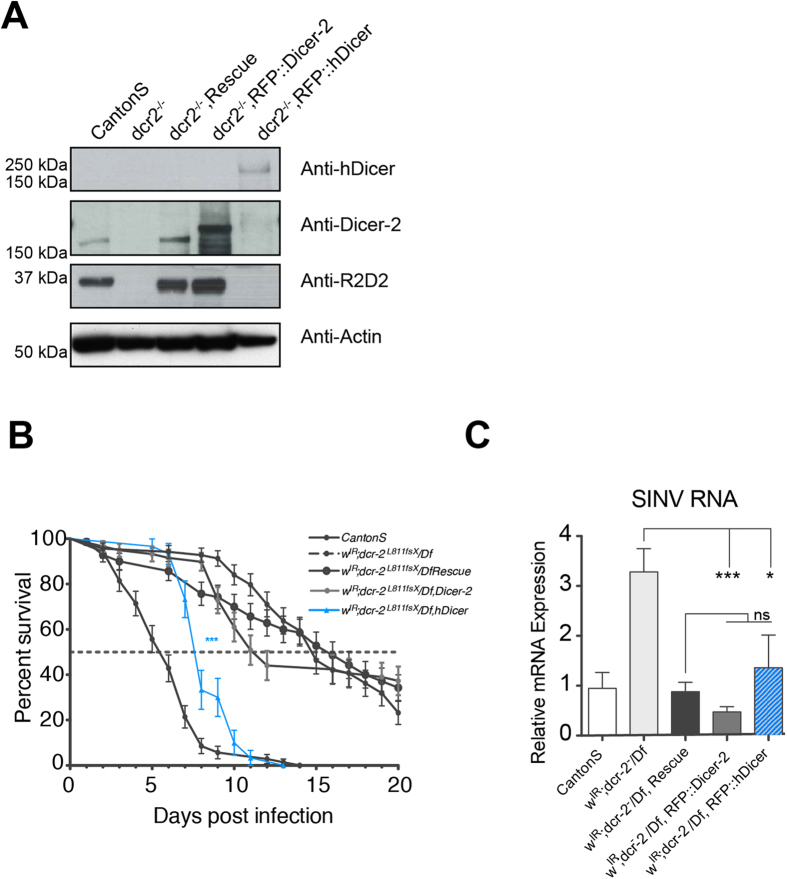
Effect of stable expression of RFP::hDicer on viral infection in *D. melanogaster.* **A**) Western blotting to measure RFP::Dicer-2, RFP::hDicer and R2D2 protein expression levels in transgenic flies. Anti-Dicer-2, anti-hDicer and anti-R2D2 antibodies were used. Anti-Actin was used as loading control. **B**) Survival curves upon SINV infection in flies. CantonS = wild-type; *w*^*IR*^*; dicer-2*^*L811fsX*^*/Df* = *dicer-2* null; *w*^*IR*^*; dicer-2*^*L811fsX*^*/Df-Rescue* = *dicer-2* null with a genomic rescue; *w*^*IR*^*; dicer-2*^*L811fsX*^*/Df*,RFP::Dicer-2 = *dicer-2* null rescued with RFP::Dicer-2; *w*^*IR*^*; dicer-2*^*L811fsX*^*/Df*,RFP::hDicer = *dicer-2* null rescued with RFP::hDicer. Each condition was compared to the survival curve of *dicer-2* null flies and wild-type. The *dicer-2* null rescued with RFP::hDicer line has significative difference with all the genetic background represented. The data represent the mean and SEM of three independent experiments. ***p < 0.001 (Log-Rank test). **C**) Viral RNA load was determined in groups of six flies 5 days post SINV infection. The data represent the mean and SEM of three independent experiments. *p < 0.05, ***p < 0.001 (t-test).

In order to test *in vivo* the intrinsic antiviral activity of hDicer in an IFN-free system, we generated transgenic flies expressing the wild-type genomic Dicer-2 (Rescue), RFP tagged Dicer-2 (RFP::Dicer-2) or hDicer (RFP::hDicer) in a *dicer-2* null (*dicer-2*^*L811fsX*^*/Df*) background (Figure S1A,B). Since Dicer-2 expression is required for the stability of R2D2^29^, we assessed R2D2 expression level in RFP::Dicer-2 and RFP::hDicer flies. R2D2 is stabilized in both RFP::Dicer-2 and Dicer-2 genomic-rescued flies, but not in RFP::hDicer flies (Fig. 1A).

We next looked whether RFP::hDicer could complement Dicer-2 function, using the white inverted repeat (w^IR^) reporter for activity of the siRNA pathway^12^. Whereas RFP::Dicer-2 expression in the *dicer-2* null mutant background rescued the eye color phenotype, expression of RFP::hDicer did not (Figure S2). Furthermore, 21-nt-long siRNAs matching the sequences of the *w*^*IR*^ transgene are present in Dicer-2 rescued flies (59950 reads), but appear to be reduced in both RFP::hDicer flies (1641 reads) and *dicer-2* null flies (550 reads).

We next explored if hDicer could rescue antiviral RNAi in *dicer-2* null mutant flies. As previously reported, *dicer-2* null flies were highly susceptible to SINV infection compared to wild-type (Canton S) controls^18^,^30^, and this phenotype could be rescued by both the wild-type genomic Dicer-2 and the RFP::Dicer-2 transgenes (Fig. 1B). Interestingly after SINV infection, expression of the RFP::hDicer transgene prolonged viability of the mutant flies, although not as efficiently as the RFP::Dicer-2 transgenes (Fig. 1B). Monitoring of the viral RNA load in the infected flies at 5 days post infection (dpi), further indicated that expression of hDicer results in lower accumulation of viral RNA (Fig. 1C). Similar results were obtained with an independent RFP::hDicer recombinant line (data not shown).

To get insight on the mechanism involved in viral restriction by hDicer, we sequenced sRNAs 5 days post SINV infection (corresponding to LT50 of *dicer-2* null background) from a pool of six flies per condition. Total reads were mapped to both *D. melanogaster* and SINV genomes with 0 mismatch and species-specific reads were kept for further analysis. The expression of cellular miRNAs remained globally unaffected in the different lines, suggesting that hDicer does not interfere with miRNA processing (Figure S3).

**Figure 2:**
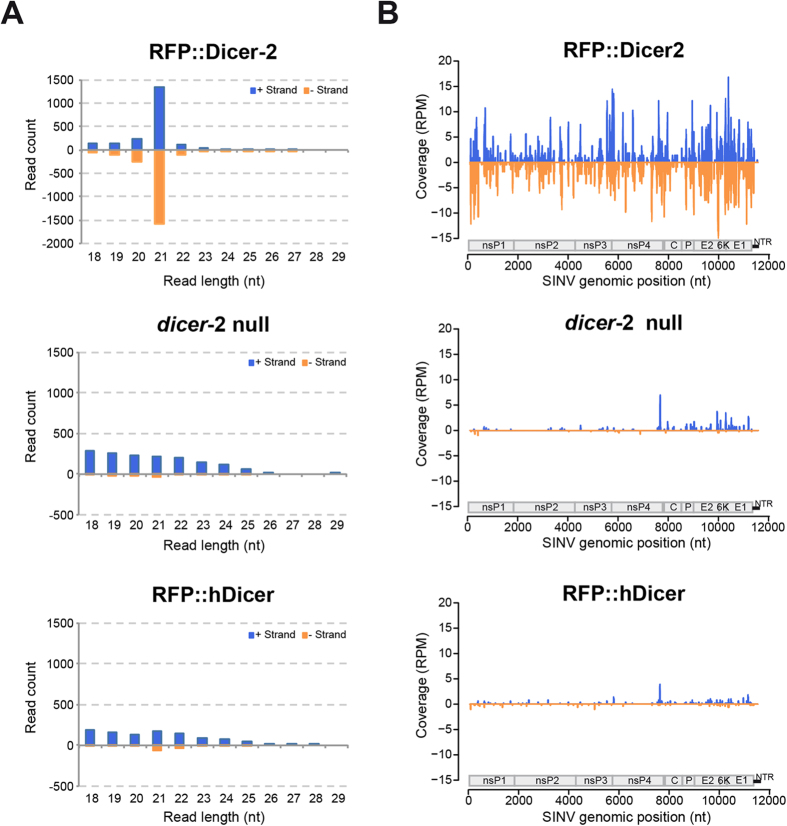
Small RNA deep-sequencing analysis of viral reads from transgenic flies. ** A)** Size distribution of viral sRNA populations. **B)** Coverage of the 21-nt viral reads was calculated and plotted as the sum of normalized reads (RPM, Reads Per Million mapped reads) in each single-nucleotide sliding window along the SINV genome. A schematic diagram represents the organization of SINV genome. The two open reading frames (ORF), which encode the nonstructural (ns) and structural proteins, are shown. The non-translated region (NTR) at the 3′ end of the virus is shown as a small black bar. Positive (+) and negative (−) strand-derived reads are shown in blue and orange, respectively.

Next, we analyzed the virus-derived sRNAs (Fig. 2). The majority of SINV-derived small RNAs in RFP::Dicer-2 flies were 21 nt in length (Fig. 2A, upper panel) and mapped in roughly equal numbers to both SINV genome and anti-genome (Fig. 2B, upper panel), as expected for Dicer-2 products generated from double-stranded viral intermediates of replication. By contrast, viral sRNAs mainly derived from the genomic (+) RNA in *dicer-2* null as well as in RFP::hDicer flies, and were at first sight not particularly enriched for a specific size (Fig. 2A, middle and lower panel). As the (+) strand is present in excess of the (−) strand in SINV infected cells, these sRNAs most likely represent degradation products, as described in mammalian cells^26^. Indeed, their genomic distribution is strongly biased towards the 3′ end of the SINV genome, corresponding to the highly expressed sub-genomic RNA encoding the structural proteins of the virus (Fig. 2B, middle and lower panel). Nonetheless, we detected significantly more 21 nt-long viral sRNAs in RFP::hDicer transgenic flies compared to *dicer-2* null ones (AC-statistic test, adjusted p-value < 0.002). Interestingly, the same statistic test applied to w^IR^ 21 nt-long sRNAs revealed that the difference between RFP::hDicer transgenic and *dicer-2* null flies was not significant.

Even though there were slightly more reads in hDicer expressing flies, we conclude that the limited amount of SINV-derived 21 nt sRNAs produced is unlikely to be relevant to interfere with viral replication. We thus hypothesized that hDicer may function in a different way to display its partial antiviral function in flies. Since viral infection also triggers an inducible immune response in *Drosophila*, we monitored expression of marker genes associated with the fly innate immune response^31^,^32^,^33^,^34^. However, we did not observe any consistent trend that could explain the increased resistance to SINV infection in *dicer-2* mutant flies rescued by Dicer-2 or hDicer transgenes (Figure S4).

### 21-nt-long viral sRNAs are produced in Dicer-2/R2D2 HEK293 cells

**Figure 3:**
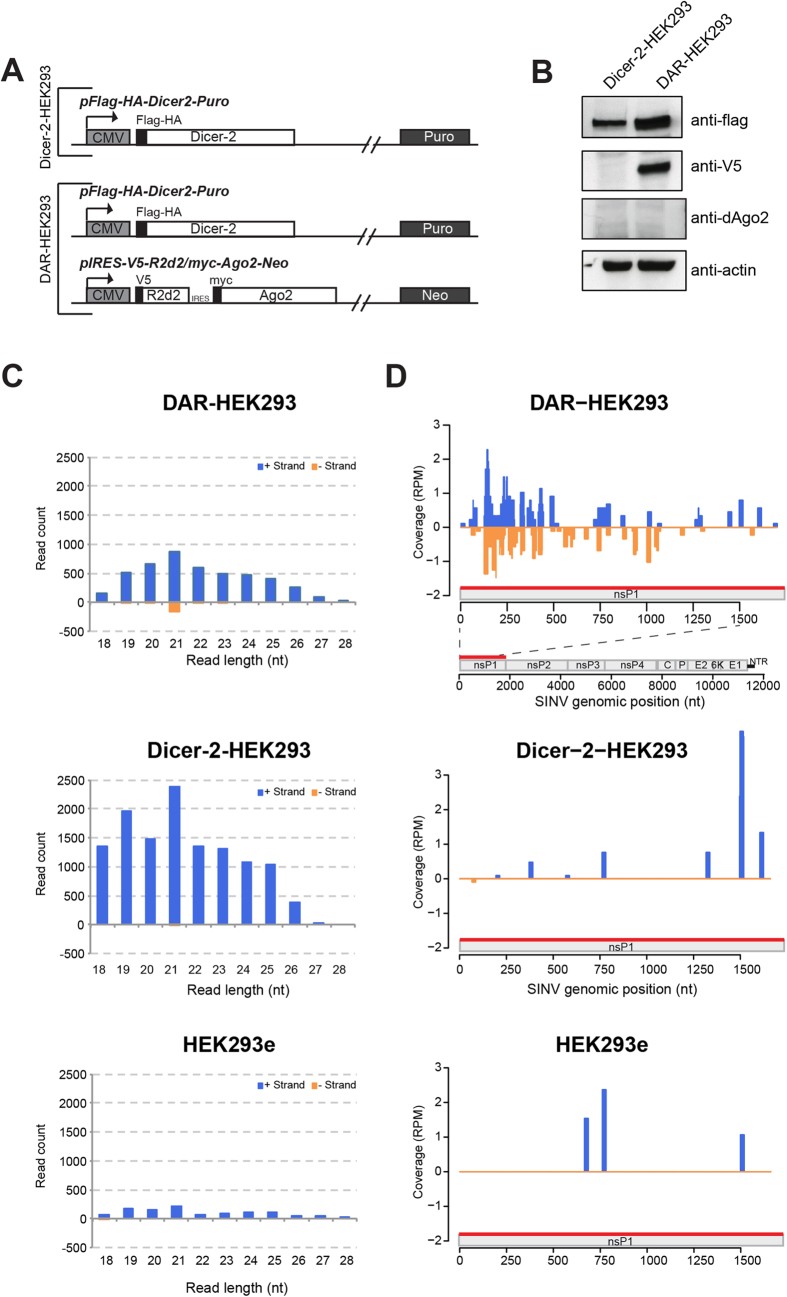
Stable expression of *D. melanogaster* RNAi components in human cell lines and deep-sequencing analysis of viral small RNAs upon SINV infection. ** A)** Schematic representation of the plasmids used in this study to generate Dicer-2 and DAR-HEK293 stable cell lines, respectively. The plasmid pFlag-HA-Dicer-2-Puro drives the expression of Dicer-2 under the control of the CMV promoter. The plasmid pIRES-V5-R2D2/myc-dAgo2-Neo drives the expression of R2D2 and dAgo2 through an IRES and is under the control of the CMV promoter. **B**) Western blotting to measure Flag-HA-Dicer2, V5-R2D2, and dAgo2 protein expression levels in HEK293 cell lines. Anti-Flag, anti-V5 and anti-dAgo2 antibodies were used. Anti-actin was used as loading control. **C-D**) Deep sequencing in SINV-infected HEK293 stable cell lines (6 hpi). **C**) Size distribution of viral sRNA populations. **D**) Coverage of the 21-nt viral reads was calculated and plotted as the sum of normalized reads (RPM, Reads Per Million mapped reads) in each single-nucleotide sliding window along the first 1500 nt of the SINV genome which correspond to nsP1 coding region (red bar). For the coverage on the entire SINV genome, see Figure S8. Positive (+) and negative (−) strand-derived reads are shown in blue and orange, respectively.

We next engineered HEK293 cells to stably express specific components of the *D. melanogaster* RNAi machinery and to test whether an antiviral RNAi pathway can function in these human cells. Cells were transfected with the Flag-HA-Dicer-2 construct, alone (Dicer-2-HEK293 cell line) or in combination with a V5-R2D2/myc-Ago2 expression plasmid (**D**icer-2-d**A**go2-**R**2D2, DAR-HEK293 cell line) or with the empty vector (HEK293e) (Fig. 3A), and stable lines were established. Expression of Dicer-2 and R2D2 was verified by western blot (Fig. 3B). However, and although R2D2 and dAgo2 are encoded on a bi-cistronic mRNA (Fig. 3A), expression of dAgo2 could not be detected either by western blot (Fig. 3B) or by immunofluorescence in DAR-HEK293 (data not shown). Of note, individual expression of Dicer-2, R2D2 or dAgo2 can be achieved by transient transfection in HEK293 cells (Fig. S5), indicating that Dicer-2 is not required to stabilize R2D2 in mammalian cells, in contrast to what happens in flies^29^.

We previously reported that virus-derived siRNA accumulation cannot be measured by deep sequencing in SINV virus infected HEK293 cells^26^. To evaluate whether Dicer-2 and R2D2 function in human cells, we cloned and sequenced sRNAs isolated from HEK293e, Dicer-2-HEK293 and DAR-HEK293 respectively, at both early (6 hours) and late (16 hours) time points of SINV infection. We verified whether the expression of Dicer-2, with or without R2D2, affected the endogenous miRNA profile in the different stable cell lines. We observed that the global profile of cellular miRNAs in each infected sample was not affected at 6 hours post infection (hpi), suggesting that Dicer-2 expression does not interfere with miRNA processing early during infection (Figure S6). This was in agreement with the recent observation that Dicer-2 does not rescue miRNA processing in hDicer-null cells^35^. However, at 16 hpi, the global miRNA profiles seem to be somewhat perturbed in Dicer-2 expressing cells (Figure S6).

We then focused our attention on the viral-derived sRNAs. Total reads were mapped to both the human and SINV genomes with 0 mismatch and species-specific reads were kept for the next analysis. Notably, we could clone more viral sRNAs in the presence of Dicer-2 both at 6 hpi (12332, 4708 and 1096 reads respectively in Dicer-2-, DAR- and empty-HEK293 cells) and 16 hpi (60734, 34413 and 25805 reads respectively in Dicer-2-, DAR- and empty-HEK293 cells). The majority of the viral reads mapped to the viral (+) genome and exhibited a broad profile of sizes (Fig. 3C), as observed in our previous work^26^. However, we could distinguish a specific accumulation of 21-nt-long reads on the SINV (−) strand only in DAR-HEK293 both at 6 and 16 hpi (Fig. 3C and S7, upper panels) This peak was neither observed in Dicer-2-HEK293 nor HEK293e cells (Fig. 3C and S7, middle and lower panels). In addition, 21-nt-long viral sRNAs coming from both strands were significantly more abundant in DAR-HEK293 cells compared to either Dicer-2-HEK293 or HEK293e cells (AC-statistic test, adjusted p-value < 0.002). The genomic distribution of the 21-nt-long viral reads along the entire viral genome was also analyzed both at 6 and 16 hpi (Figure S8). The 21-nt sRNAs mainly originate from the first 1000 nt of SINV genome at 6 hpi (Fig. 3D, upper panel). Those 21-nt-long reads are still present in the beginning of the SINV genome at 16 hpi in DAR-HEK293 cells which co-expressed Dicer-2 and R2D2, but their distribution tends to spread along the genome compared to the 6 hpi (Figure S8, upper panels).

Finally, we also verified that the endogenous hDicer did not influence the production of the 21 nt sRNA population by knocking it down with siRNAs in both HEK293e and DAR-HEK293 cells and performing small RNA cloning and sequencing after SINV infection at 6 hpi (data not shown). No significant difference in the number of viral reads was observed in these conditions (AC-statistic test).

**Figure 4:**
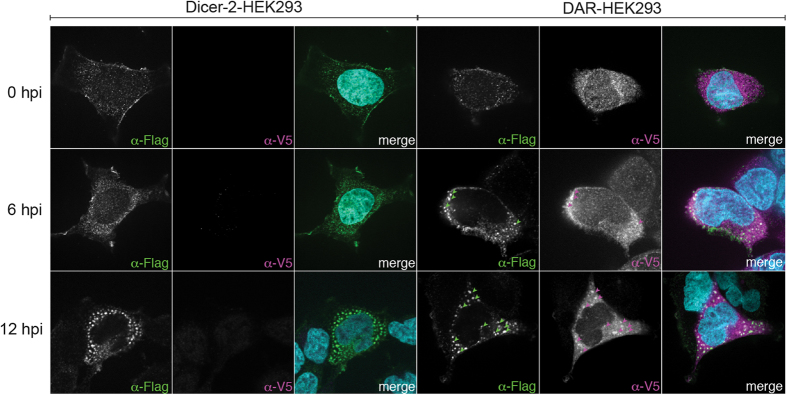
Dicer-2 and R2D2 localization in human stable cell lines upon viral infection. Dicer-2-HEK293 and DAR-HEK293 stable cell lines were immunostained with anti-Flag and anti-V5 antibodies, which recognize Flag-Dicer-2 and V5-R2D2, respectively (see also Figure S9 for dsRNA staining). Immunostaining was performed at 0, 6, 12 hours post SINV infection (MOI 1). Green and magenta arrows highlight the colocalization of Dicer-2 and R2D2 in DAR-HEK293 cells during SINV infection.

In flies, Dicer-2 and R2D2 co-localize in the cytoplasm^36^ and R2D2 is responsible for Dicer-2 loca-lization in the so-called D2 bodies, presumably together with siRNA duplexes prior to their loading in dAgo2. We therefore analyzed the localization of HA-Dicer-2 and V5-R2D2 by immunofluorescence (IF) before and after SINV infection in the stable cell lines (Fig. 4 and S9). All cell lines show a strong accumulation of dsRNA in the time-course of SINV infection (Figure S9). In mock-infected Dicer-2-HEK293 cells, Dicer-2 displays a punctuated cytoplasmic staining. Interestingly, the clear presence of granules was detectable in the cytoplasm of ~2% of the cells at 12 hpi (Fig. 4, left panels). Dicer-2 and R2D2 are present in discrete punctate granules in mock-infected DAR-HEK293 cells, without co-localizing. However, discrete overlapping granules containing both Dicer-2 and R2D2 start to appear at 6 hpi and are well detectable at 12 hpi in ~11% of the infected cells (Fig. 4, right panels). These results show that, in HEK293 cells, Dicer-2 and R2D2 co-localize exclusively during viral infection and that Dicer-2 is required to form the discrete cytoplasmic granules in the presence of SINV.

### Dicer-2 interferes with IFN signaling in human cells

**Figure 5:**
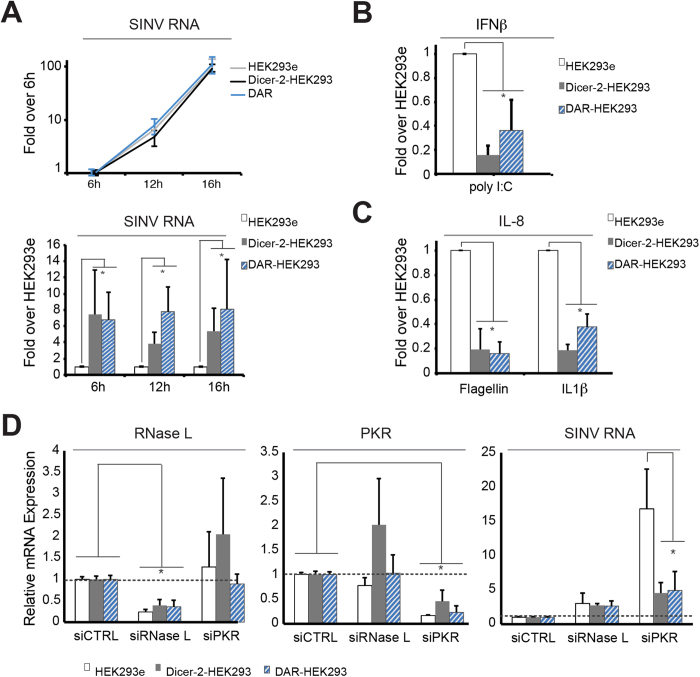
Stable expression of fly Dicer-2 in mammalian cells does not impair viral infection but compete with the IFN response. **A**) Viral RNA load in HEK293e, Dicer-2- and DAR-HEK293 stable cells infected with SINV (MOI 0.01) was determined by RT-qPCR at 6, 12 and 16 hpi. The results were normalized to the 6 hpi time point (upper panel) or to the HEK293e condition (lower panel). **B**) Activation of the IFNβ response upon dsRNA treatment in HEK293e, Dicer-2- and DAR-HEK293 cells. Cells were treated with poly I:C (20 μg/ml) and IFNβ mRNA was measured by RT-qPCR. The results obtained were normalized to HEK293e cell line. **C**) Activation of the IL-8 response upon bacteria or cytokine treatment in HEK293e, Dicer-2- and DAR-HEK293 cells. Cells were treated with Flagellin (100 ng/μl) or IL-1β and IL-8 mRNA was measured by RT-qPCR. The results obtained were normalized to HEK293e cell line. **D**) HEK293e, Dicer-2- and DAR-HEK293 cells were treated with siRNAs against RNase L, PKR or negative control (CTRL) and infected for 16 hours with SINV (MOI 0.01). Relative RNase L mRNA, PKR mRNA and SINV RNA levels were determined by RT-qPCR. The expression level of each gene is normalized to the siCTRL condition. All RT-qPCR experiments were normalized to actin-b mRNA expression and represent the mean and standard deviation of at least three independent experiments. ns (non-significant), *p < 0.05 (*t* test).

We next tested whether the expression of Dicer-2 and R2D2 in human cells could confer resistance to viral infection. Although viral RNA increased similarly in the different stable cell lines at 6, 12 and 16 hpi (Fig. 5A, upper panel), the viral load was significantly higher in Dicer-2-HEK293 and DAR-HEK293 cells compared to HEK293e (Fig. 5A, lower panel), a difference that was already detectable between 2 and 3 hpi (data not shown), suggesting a defect in the endogenous antiviral immune pathway already at the beginning of the infection. A similar positive effect on SINV was observed with an independent clone of Dicer-2-HEK293 stable cells (data not shown).

We hypothesized that Dicer-2 could compete, instead of synergizing, with the IFN pathway. To test this hypothesis, we measured the impact of Dicer-2 expression on the induction of IFNβ expression. SINV infection is notably known to induce a strong phosphorylation of PKR, which leads in turn to a translational shutdown^37^. It is therefore difficult to measure induction of the IFNβ promoter in these conditions^38^. For this reason, we measured the endogenous IFNβ expression by RT-qPCR upon polyI:C treatment. At 6 h post-transfection, *IFNβ* mRNA induction was strongly reduced both in Dicer-2 and DAR-HEK293 cells (Fig. 5B). In order to confirm these findings, we also transfected an IFNβ promoter-luciferase reporter in the three stable cell lines and measured luciferase activity upon challenge with polyI:C. Whereas polyI:C stimulation resulted in a robust induction of IFNβ promoter in the control HEK293e cell line, Dicer-2- and DAR-HEK293 cells displayed markedly reduced levels of induction of the reporter (Figure S10). Both at the levels of the endogenous *IFNβ* mRNA and with the luciferase reporter, the drop in the induction was milder in DAR cells compared to Dicer-2 cells. This suggests that Dicer-2 interferes with IFNβ transcriptional activation and that the presence of R2D2 might modulate this regulatory effect.

We also looked at the effect of non-nucleic acids based inducers of innate immunity such as bacterial flagellin or interleukin-1β, which activate respectively TLR-5^39^ and IL1R^40^. The analysis of *IL8* mRNA by RT-qPCR revealed that for both inducers, Dicer-2 and DAR-HEK293 cells also displayed a reduced level of activation compared to control cells (Fig. 5C). This latest result indicates that the expression of Dicer-2 in HEK293 cells globally perturbs innate immunity.

To further investigate the crosstalk between the Dicer-2 protein and the innate immune pathway, we down-regulated PKR and RNase L, two key proteins involved in viral RNA sensing and/or processing. Cells were infected with SINV for 16 hours following PKR or RNase L knockdown and the viral load was measured in the different stable cell lines (Fig. 5D). Silencing of RNase L had the same effect in all cell lines, i.e. a 2- to 3-fold increase in viral RNA accumulation. However, the strong positive effect of PKR knockdown on SINV RNA accumulation was severely reduced in Dicer-2 and DAR-HEK293 cells compared to control HEK293e cells (Fig. 5D, right panel). This suggests that Dicer-2 expression, independently of the presence of R2D2, alters dsRNA sensing and signal transduction in SINV-infected human cells.

## Discussion

While RNA-based silencing represents the main response to viruses in insects, vertebrates have evolved a complex and robust protein-based line of defense against viral infection, which ultimately relies on signal transduction by IFN and the expression of hundreds of antiviral IFN-stimulated genes (ISGs). Recent observations in mESCs and suckling mice^24^,^25^ support the idea that RNAi function is conserved during evolution and contributes to the antiviral response also in vertebrates. However, the role of Dicer in RNA silencing against viruses in IFN-competent human cells has proven difficult to be formerly defined.

In our study, we conduct an original investigation of the fly and human antiviral defense and examine the function of human (h)Dicer and *Drosophila* (d)Dicer-2 in heterologous systems.

In order to challenge both systems with the same virus, we chose the arbovirus SINV. In addition to its ability to infect both insects and mammals, the production of SINV-derived small RNAs has been already well characterized in both systems^18^,^26^. We took advantage of high-throughput sequencing technology to dig into the small RNA repertoire generated in our experimental settings. However, it is important to keep in mind that our deep-sequencing data represent a qualitative analysis and cannot be taken as a quantitative and absolute result.

Our data reveal that the expression of hDicer in *D. melanogaster* partially rescued the lack of Dicer-2 in terms of antiviral defense against SINV. Yet, very few virus-derived siRNA-like molecules are generated in hDicer-rescued flies compared to Dicer-2-rescued flies. In addition, their size (21nt) was smaller than classical hDicer products (22-23nt). This may reflect a difference in folding of this multidomain protein at 25 °C, possibly related to the lack of TRBP or PACT in this experimental setting. Alternatively, we cannot rule out that this difference in size reflects a preferential loading and stabilization of this size range in the RISC.

Even though the low accumulation of virus-derived small RNA is probably not sufficient to silence the virus, the effect of hDicer expression on fly survival and antiviral resistance is remarkable. It might be explained by the possibility that hDicer also restricts viral infection by other means. For example, it could bind to viral RNAs and interferes with viral replication.

Altogether, these findings indicate that the human Dicer has no intrinsic inability to function in antiviral immunity, but is not efficient in producing siRNAs in *Drosophila*.

We also tested the capacity of IFN-competent differentiated human cells to support antiviral RNAi. We thus generated a human cell line expressing *D. melanogaster* Dicer-2, with or without its cofactor R2D2. The accumulation of SINV-derived 21-nt-long siRNA-like molecules is usually undetectable in human somatic cells, but in the presence of Dicer-2 and R2D2 we could specifically clone and sequence 21-nt long viral RNAs derived from both SINV genome and anti-genome. This suggests that Dicer-2 requires R2D2 for the proper recognition and processing of a viral RNA target in this cellular system. Furthermore, our results indicate that there is no intrinsic restriction factor preventing these human cells from producing viral siRNAs.

The stably expressed Dicer-2 and R2D2 do not co-localize in non-infected HEK293 cells. However, Dicer-2/R2D2 granules appear in the cytoplasm upon viral infection and are reminiscent of the previously described D2 bodies in the *D. melanogaster* S2 cell line^36^. However, in the case of *Drosophila* cells, these bodies are constitutive, which might reflect the presence of endogenous dsRNAs (e.g. persistent viral infections^41^,^42^) recruiting Dicer-2 and R2D2. Our heterologous system could therefore provide a useful tool to investigate the mechanisms at play for the recruitment of antiviral RNAi components on viral RNA substrates.

One of the key findings of this study is the observation that the presence of a functional machinery that cleaves viral RNA molecules into siRNAs is not sufficient to confer a protective antiviral effect in somatic human cells. On the contrary, our results indicate that the presence of Dicer-2 is rather beneficial for the virus in this setup. This can be visualized by an increase in virus accumulation, which probably also explains the observed changes in cellular miRNA profiles in DAR and Dicer-2-HEK293 cells at later time points. We show that this is most likely due to an incompatibility with the IFN pathway. In particular, the presence of Dicer-2 has a negative effect on IFNβ induction by poly:IC and IL-8 induction by flagellin and IL1β, and seems to compete with dsRNA-sensing factors such as PKR. We hypothesize that the competition could occur at the level of RNA sensing by these factors. Some indications on the reciprocal inhibition of RNAi and IFN pathway have been previously reported. For instance, viral infection or polyI:C treatment induces pADP-ribosylation and inhibition of RISC-associated proteins^43^. Also, it has been shown that interferon-induced proteins, such as IFNβ and OAS1, were strongly up-regulated in hDicer-knockdown cells^44^.

**Figure 6:**
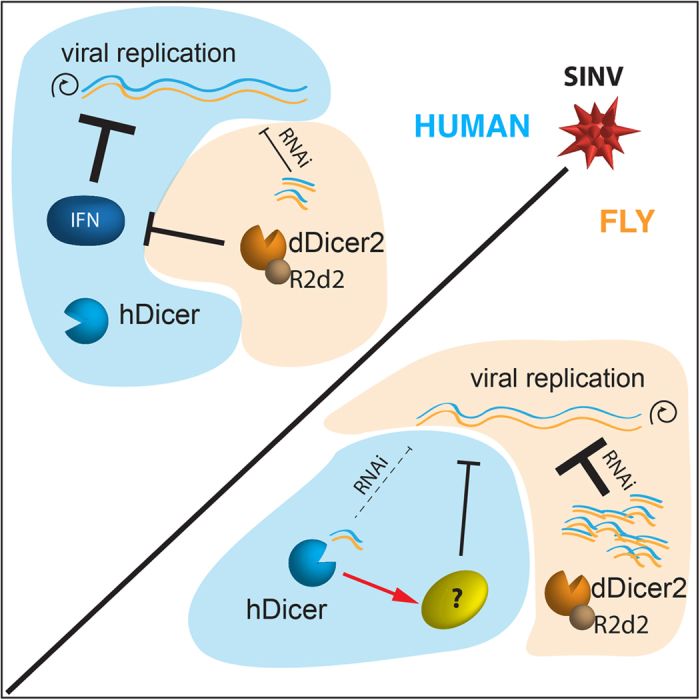
Hypothetical working model for hDicer and dDicer-2 in heterologous systems upon SINV infection. In human cells, the IFN pathway plays a major role to counteract SINV infection, while hDicer has not been demonstrated to act against the virus. Expression of *D. melanogaster* Dicer-2 and its cofactor R2D2 allows the production of viral 21-nt –long small RNAs but, instead of conferring resistance Dicer2 antagonizes the IFN-based antiviral immunity against the virus. In flies, Dicer2/R2D2 complex plays a key role in RNAi to silence SINV. Expression of hDicer only partially rescues RNAi-mutant flies for the production of viral siRNAs, but contributes to the antiviral response against SINV by an as yet uncharacterized mechanism.

In conclusion, our work sheds a new light on the intrinsic role of Dicer proteins in antiviral defense and brings new insights into the debate on RNAi function in mammalian somatic cells. According to our working model (Fig. 6), human Dicer would be lowly active to process dsRNAs in flies, but would play a role in antiviral activity through unknown pathways. In human cells, the fly Dicer-2 would be competent for dsRNA processing only in combination with R2D2, but rather than conferring antiviral resistance, would compete with the innate immune response. Thus, we could speculate that the antiviral activity of endogenous hDicer could have been restrained in human somatic cells, because of a functional incompatibility with the IFN response.

## Materials and Methods

### Generation of transgenic flies

We generated independent transgenic lines both for RFP::Dicer-2 and RFP::hDicer. Detailed cloning procedure is available in supporting information. Transgenic constructs consisting of N-term RFP::Dicer-2 and RFP::hDicer are both under the control of the poly-ubiquitin promoter. Transgenic lines were generated by standard methods in *w*^*1118*^ genetic background. All transgene insertions on the Drosophila genome were verified by inverse PCR (http://www.fruitfly.org/about/methods/inverse.pcr.html) (Figure S1). Recombination with Df(2R)BSC45 and crossing with *dicer-2*^*L811fsX*^*/CyO* were performed.

Hemizygous *dicer-2* null mutant flies correspond to the following genotype [*dicer-2*^*L811fsX*^*/Df(2R)BSC45*]. Flies with the genomic rescue of the *dicer-2* gene [*dicer-2*^*L811fsX*^*/Df(2R)BSC45,* Dcr-2-Rescue]^45^ are also called “Rescue”. CantonS (wild type) flies were used as control (see Figure S1). All the flies used in this study have the *w*^*IR*^ transgene on the X-chromosome^12^.

### Generation of HEK293-derived stable cell lines

HEK293 cells were transfected with pFlag-HA-Dicer-2-Puro and selected for about 6 weeks with puromycin (3 μg/ml). Resistant Dicer-2-HEK293 colonies were established and subsequently maintained under puromycin selection. The DAR-HEK293 stable cell line was generated by transfection of pIRES-V5-R2D2/myc-Ago2-Neo in Dicer-2-HEK293 cell line and selection with both puromycin and G418 at the working concentration of 3 μg/ml and 1 mg/ml, respectively. The HEK293e cell line was generated using the empty pDEST Flag-HA vector. Detailed cloning procedure is available in supplementary information.

### Small RNA cloning and sequencing

A pool of either six SINV-infected *dicer-2* null flies, or RFP::hDicer flies or RFP::Dicer-2 flies was collected for RNA extraction at 5 days post-infection for small RNA library preparation with SINV at 2500pfu. Additionally, HEK293e, Dicer-2-HEK293 and DAR-HEK293 cells were infected with SINV (MOI 0.01) for 6 hrs and collected for RNA extraction. Before being infected, HEK293e and DAR-HEK293e cells were transfected with either control siRNAs or hDicer siRNAs. Non-transfected HEK293e, Dicer-2-HEK293 and DAR-HEK293 cells were also infected with SINV (MOI 0.01) for 16 hrs before RNA extraction. Small RNA cloning was conducted with 5-10 μg of total RNA as previously described^46^. Size fractionation was performed excluding the 2S rRNA in the fly samples. High-throughput sequencing was performed at the IGBMC Microarray and Sequencing platform, Illkirch, France, using an Illumina HiSeq 2500 instrument with a read length of 50 nt.

### Bioinformatics analysis of deep sequencing data

Sequencing reads were preprocessed and annotated using a set of custom Python scripts pipelining different tools. The Dustmasker program^47^ and FASTX-Toolkit (http://hannonlab.cshl.edu/fastx_toolkit) were first applied to filter out low complexity reads and remove instances of the 3′ adapter. Degenerate bases incorporated during the library preparation protocol were then trimmed and remaining reads were further size-selected by keeping only the 18- to 32-nt long ones. In the case of human libraries, exogenous siRNA sequences were also excluded before further analysis. Preprocessed reads were then mapped simultaneously to the host (either *Drosophila melanogaster* r5.54 – Flybase, or *Homo sapiens* hg19 – UCSC repository) and the pathogen (Sindbis virus NC_001547.1 – RefSeq database) genomes using Bowtie 1.0.0^48^. In the case of fly libraries, small RNA reads were also mapped to the white-inverted repeats, *w*^*IR*^ transgene, as previously shown^17^. Initially, only alignments of reads with 0 mismatch were recorded and reads that could map to more than 50 loci were discarded.

For each library, small RNAs deriving solely from SINV, in either sense or antisense orientation, were computationally extracted and profiled based on their length distribution and their coverage along the viral genome. The latter was calculated and plotted as the sum of normalized reads (RPM, Reads Per Million mapped reads) in each single-nucleotide sliding window along the SINV genome using R/Bioconductor and the GenomicAlignments package. To detect differentially expressed viral reads of 20, 21, 22, 23 and 24 nt between pairs of libraries, we applied the Audic and Claverie (AC) statistic test^49^ through the web tool IDEG6 developed by Romualdi *et al.*^50^. The p-value threshold was set to 0.01 and the Bonferroni method was applied for multiple testing corrections. An adjusted p-value < 0.002 was considered significant. The same approach was used to deal with reads deriving from the *w*^*IR*^ transgene.

In order to establish the host miRNA expression profiling, host-specific reads presenting with up to 2 mismatches in total with no more than 1 mismatch in their first 15 nucleotides were taken into account. Fly or human miRNAs (miRBase Release 20^51^) were annotated in each library using BEDTools 2.16.2^52^. During the quantification process, multiple mapped reads were weighted by the number of mapping sites in miRNAs. To visually explore miRNA expression profiles in each library set, we produced heatmaps showing the expression data of the 100 most highly expressed miRs, after regularized log transformation, as recommended in the DESeq2 package^53^.

## Additional Information

**How to cite this article**: Girardi, E. *et al.* Cross-species comparative analysis of Dicer proteins during Sindbis virus infection. *Sci. Rep.* **5**, 10693; doi: 10.1038/srep10693 (2015).

**Accession codes**: Sequencing data were deposited in NCBI’s Gene Expression Omnibus with the accession number GSE62934.

## Supplementary Material

Supporting Information
