# Supplementary material for: Cross-species comparative analysis of Dicer proteins during Sindbis virus infection
Source: Sci Rep. 2015 May 29;5:10693. doi: 10.1038/srep10693 (PMC4448662; doi:10.1038/srep10693)
Supplement: Supporting Information [file srep10693-s1.pdf]

Cross-species comparative analysis of Dicer proteins during Sindbis virus infection

Erika Girardi[1,3], Mathieu Lefèvre[1], Béatrice Chane-Woon-Ming[1], Simona Paro[2], Bill Claydon[2], Jean-Luc Imler[2], Carine Meignin[2,*], Sébastien Pfeffer[1,*]

SUPPLEMENTARY INFORMATION

SUPPLEMENTARY METHODS

Constructs used in the study

A PCR fragment corresponding to the whole Dicer-2 cDNA sequence was amplified from Dicer-2 full-length pOT2-cDNA clone (derived from SD11113 clone) using the primers indicated in OLIGO LIST. Dicer-2 was cloned into the pENTR/D-TOPO Gateway entry vector using the pENTR directional TOPO cloning kit (Invitrogen) for N-terminal fusion. pFRT/TO/FLAG/HA-DEST DICER vector (Addgene) was used as starting template to generate by recombination pDONOR-ZEO-hDicer.

The Dicer-2 and hDicer cDNA were then transferred to Drosophila transgenic destination vector pURW (a kind gift from Jean-René Huynh's laboratory, DGRC#1282) by LR recombination using the Gateway LR clonase II enzyme mix (Invitrogen) and used for transgenic flies generation.

To obtain pFlag-HA-Dicer-2-Puro, we recombined the pDONOR-Dicer-2 with the destination vector pDEST Flag-HA vector using the Gateway cloning system. To obtain pIRES-V5-R2D2/myc-Ago2-Neo, R2D2 and Ago2 genes were amplified by

PCR (See OLIGO LIST) and cloned into pIRES vector (cat# 631605 Clontech). pFlag-HA-R2d2 and pFLAg-HA-Ago2 have been generated as for pFlag-HA-Dicer2 and only used for transient transfection experiments.

Fly maintenance and infection conditions

Flies were fed on standard cornmeal–agar medium at 25 ℃. All fly lines were tested for Wolbachia infection and cured whenever necessary. Infections were performed with 3- to 5-days old adult flies by intrathoracic injection (Nanoject II apparatus; Drummond Scientific) with viral particles. Sindbis viral stock was prepared in 10 mM Tris-HCl, pH 7.5 [SINV $5x10^8$ PFU/ml]. Injection of the same volume (4.6 nL) of 10 mM Tris-HCl, pH 7.5, was used as a control. Infected flies were then incubated at 25°C, and monitored daily for survival, or frozen for RNA isolation and virus titration at 5 days post infection (dpi).

Viral stocks, cell culture and virus infection

TE3'2J infectious clone containing SINV genomic sequence was linearized with XhoI and used as a substrate for *in vitro* transcription using mMESSAGE mMACHINE capped RNA transcription kit (Ambion) following the manufacturer's instructions. Sindbis viral stock was prepared in BHK21 hamster kidney cells and titrated by plaque assay. HEK293 cells were maintained in Dulbecco's modified Eagle medium (DMEM, Gibco) supplemented with 10% fetal bovine serum (FBS, Clontech) in a humidified atmosphere of 5% CO2 at 37°C. Cells were infected with SINV at MOI 0.01 (except when indicated differently) and samples were collected at different time points as indicated in the figure legends.

RNA Extraction and Real-Time PCR analysis

For the human samples, total RNA was isolated using TRI-Reagent (Ambion, AM9738) and 2 μg of RNA were DNaseI treated and reverse transcribed with SuperScript III (Invitrogen, Ref: 18080-080) according to manufacturer's instructions. Quantitative real-time PCR was performed using Maxima SYBR Green (Fermentas, #K0253), on a CFX96 Touch™ Real-Time PCR Detection System (Biorad). Gene expression was normalized to beta actin.

Total RNA from infected flies was isolated using TRI Reagent RT bromoanisole solution (MRC) and reverse transcribed using iScript cDNA Synthesis Kit (Bio-Rad) on the T3000 Thermocycler (Biometra). Quantitative real-time PCR was performed using the iQ Custom SYBR Green Supermix Kit (Bio-Rad) on the CFX384 Real-Time System (Bio-Rad). Gene expression was normalized to RP49.

All the primers used are listed in the OLIGO LIST.

Statistical analysis

An unpaired two-tailed Student t-test was used for statistical analysis of data with GraphPad Prism (GraphPad Software). The p-values < 0.05 were considered statistically significant. Survival curves were plotted and analyzed by log-rank test using GraphPad Prism (GraphPad Software).

RNAi-mediated protein depletion

SiRNA transfection was performed using the reverse transfection method. Briefly, siRNAs (100 nM, final concentration) were incubated with lipofectamine 2000 (Invitrogen) as described by the manufacturer. Transfection mix was incubated to the cells in suspension. Two days later, transfected cells were infected with SINV MOI

0.01. Sixteen hours post-infection, cells were lysed for RNA analysis. SiRNAs used were ON-TARGET plus Smart Pool siRNAs (Dharmacon) targeting human RNaseL (L-005032-01), PKR (L-003527-00), RIG-I (L-012511-00), MDA5 (L-013041-00), hDicer (L-003483-00) and a siRNA control (D-001810-10).

Western-blot and immunostaining analysis

Proteins were extracted from 10 females of each genotype and homogenized in 200 μl of lysis buffer (30 mM Hepes, 150 mM NaCl, 2 mM Mg(OAc)2, 5 mM DTT, 0.1% NP40 and protease inhibitor 'cocktail' (cOmplete Mini; Roche Diagnostics)) with the Precellys®24 Dual, according to the manufacturer's instructions. Proteins were quantified by the Bradford method and 50 μg of total protein extract was loaded on 4-20% Mini-PROTEAN® TGX™ Precast Gels (Biorad). After transfer onto nitrocellulose membrane, equal loading was verified by Ponceau staining. Membranes were blocked in 5% milk. The membrane was probed with the following antibodies: anti-Dicer2 (1:1000, anti-PAZ domain, as indicated in Fig S1A), anti-hDicer (1:1000, Bethyl,#A301-936A), anti-R2D2 (1:2000, [1]), anti-V5 (1:5000, Abcam, ab9116), anti-drosophila Argonaute 2 (kind gift of Prof. M. Siomi), anti-HA (1:10000, Sigma, H9658) and anti-Actin clone 4 (1:5000, Millipore #MAB1501R). Detection was performed using Chemiluminescent Substrate (Amersham). Immunofluorescence experiments were carried out on cells infected with SINV-GFP (MOI 1) to guarantee that each cell was infected with 1 viral particle. Given the observed cell death at 16hpi using MOI 1, we chose the 12hpi time point for this experiment.

Mouse anti-FLAG (1:500, Sigma F-3165), rabbit anti-V5 (1:500, Abcam ab9116), mouse Anti-dsRNA J2 (1:200, ESC- 10010200), Alexa Fluor® 488 anti-mouse (1:500, Molecular Probes® A-21202), Alexa Fluor® 568 anti-rabbit (1:500,

Molecular Probes® A10042) and DAPI to visualize cell nuclei. Images were taken using Spinning disc Zeiss. ImageJ was used to analyze the cells.

IFNβ promoter-luciferase assay and IFNβ mRNA induction assay

The promoter region of the human IFNβ gene was amplified from genomic DNA and cloned upstream of the firefly luciferase gene in the pGL4-basic vector. The resulting plasmid, designated pGL4-IFNβ-luc, was transfected in the HEK293 stable cell lines in 96 well plates using lipofectamine 2000 (Invitrogen) according to the manufacturer instructions. Two days post-transfection, cells were incubated with free opti-MEM (untreated) or dsRNA poly I:C (Invivogen, tlrl-picw) at the indicated concentrations.

Twenty-four hours after treatment, cells were lysed and IFNβ promoter activity was determined using a luciferase assay (Promega, Dual Luciferase Reporter Assay, E1960) with a luminometer (Promega, GloMax® Multi Detection System). Absolute firefly luciferase activity was normalized for transfection efficiency using the untreated cells.

For the detection of the IFNβ and IL8 mRNA induction, cells were transfected with either poly I:C (20μg/mL) using with lipofectamine 2000 (Invitrogen) as described by the manufacturer or treated with Flagellin (100ng/mL; Flagellin from S. typhimurium, InvivoGen) or IL-1β (10ng/mL; Recombinant human interleukin-1beta, InvivoGen). Cells were lysed 6 hours and total RNA was extracted and retrotranscribed as mentioned above.

SUPPLEMENTARY FIGURE LEGENDS

Figure S1: **Generation of RFP::hDicer and RFP::Dicer-2 transgenic flies**. **A**) Schematic representation of Dicer-2 and hDicer domain architecture. The Helicase domain contains the DExD/H, DExD/DExH box helicase domain and HELiCc, helicase conserved C-terminal domain. dsRBD, double-strand RNA binding domain; PAZ, PAZ domain; RIIIa and RIIIb, ribonuclease III domains. Platform-PAZ-connector helix was defined by [2]. L811fsX represents the null allele of *dicer-2* used in this study. Peptide 849-866 was used to produce the Dicer-2 antibody. **B**) Genetic of Dicer-2 and hDicer transgenic lines. *Dicer-2* gene is localized in the 2nd-chromosome in 2R, 54C10. The Deficiency Df(2R)BSC45 covers the cytologic map from 2R, 54C8 to 2R, 54E7. The Dicer-2 genomic rescue contains the full *dicer-2* gene. The lower panels schematically represent the cytologic position of each transgene used.

Figure S2: Effect of RFP::hDicer and RFP::Dicer-2 expression on *D. melanogaster* endogenous siRNA pathway. Eye color of the following genotypes: (1) CantonS (wt), (2), wIR; dicer-2L811fsX/Df, (3) wIR; dicer-2L811fsX/Df-Rescue, (4) wIR; dicer-2L811fsX/Df-RFP::Dicer-2, (5) wIR; dicer-2L811fsX/Df-RFP::hDicer.

Figure S3: **Cellular miRNA profiling in SINV-infected transgenic flies**. The heatmap shows hierarchical clustering of the different samples and of the 100 most abundant miRNAs in each sample on the basis of their expression profile. High relative miRNA expression (log2-transformed) is indicated by blue shades, low expression by green shades.

Figure S4: **Effect of RFP::hDicer expression on *Drosophila melanogaster* innate immune response.** Relative mRNA level of vago (Dicer-2-dependent), diptericin (IMD pathway), drosomycin (Toll pathway), TotM (Jak/STAT pathway) and vir-1 (viral infection related) compared to the housekeeping gene rp49 in flies upon SINV infection. The expression level of each gene is normalized to the genotype wIR; dcr2-/Df, Rescue. Data are representative of at least three independent experiments (average and SEM) with 6 flies of each genotype. T-test was performed between the control genotype wIR; dcr2-/Df, Rescue and Dicer-2 null (wIR; dcr2-/Df) or RFP::Dicer-2 (5A) and RFP::hDicer (1A and 1D). ns (non-significative), *p<0.05, **p<0.01, ***p<0.001. All experiments were performed at 25°C and flies were collected 5 days post-infection by SINV at 2500 plaque forming units (pfu).

Figure S5: **R2D2, Dicer2 and dAgo2 can be individually expressed in HEK293 cells. Left panel** Schematic representation of the plasmids used for transient transfection in HEK293 cells. The plasmid pFlag-HA-Dicer-Puro, pFlag-HA-Ago2-Puro and pFlag-HA-R2D2-Puro drives the expression of Dicer-2, Ago2 and R2D2, respectively. **Right panel** Western blotting to measure Flag-HA-Dicer2, -R2D2, and -dAgo2 protein expression levels in HEK293 cell lines. Anti-HA antibody was used. Ponceau staining was used as loading control. *, ** and *** indicate the expected size for Dicer2, Ago2 and R2d2 respectively. – indicates the mock transfected control.

Figure S6: **Cellular miRNA profiling in SINV infected human cell lines**. The heatmap shows hierarchical clustering of the different samples and of the 100 most abundant miRNAs in each sample on the basis of their expression profile. High

relative miRNA expression (log2-transformed) is indicated by blue shades, low expression by green shades.

Figure S7: **Deep sequencing of viral sRNAs in SINV infected HEK293 stable cell lines at 16 hours post infection**. Size distribution of SINV-derived sRNA populations.

Figure S8: Comparison of 21-nt long viral sRNAs in SINV infected HEK293 stable cell lines at both 6 and 16 hours post infection. Coverage of the 21-nt viral reads was calculated and plotted as the sum of normalized reads (RPM, Reads Per Million mapped reads) in each single-nucleotide sliding window along the SINV genome. A schematic diagram represents the organization of SINV genome. The red bar corresponds to the first 1500 nt (nsp1) of SINV genome, as shown in Figure 3 and S7. Positive (+) and negative (-) strand-derived reads are shown in blue and orange, respectively.

Figure S9: Accumulation of dsRNA during Sindbis infection. HEK293e, Dicer-2-HEK293 and DAR-HEK293 stable cell lines were immunostained with anti-dsRNA J2 antibody (magenta). Immunostaining was performed at 0, 6, 12 hours post SINV or (MOI 1). DAPI staining is shown in blue.

Figure S10: Activation of the IFNβ promoter in HEK293e, Dicer-2- and DAR-HEK293 cells. Cells were transfected with pGL4-IFN-β-firefly luciferase constructs and were treated with polyI:C (5, 10, 20 μg/ml). Relative luciferase was measured

compared to unchallenged condition. All observed differences were significant (t-test $p<0.05$).

SUPPLEMENTARY REFERENCES

1. Nishida, K. M. *et al*. Roles of R2D2, a Cytoplasmic D2 Body Component, in the Endogenous siRNA Pathway in Drosophila. *Molecular Cell* **49,** 680–691 (2013).
2. MacRae, I. J. Structural Basis for Double-Stranded RNA Processing by Dicer. *Science* **311,** 195–198 (2006).

SUPPLEMENTARY TABLE

List of oligonucleotides used in this study

qRT-PCR

b-Actin fw	TTTGAGACCTTCAACACCCCA
b-Actin rv	TTTCGTGGATGCCACAGGA
diptericin fw	GCTGCGCAATCGCTTCTACT
diptericin rv	TGGTGGAGTGGGCTTCATG
drosomycin fw	CGTGAGAACCTTTTCCAATATGATG
drosomycin rv	TCCCAGGACCACCAGCAT
EIF2AK2(PKR) fw	TGGAAAGCGAACAAGGAGTAAG
EIF2AK2(PKR) rv	CCAAAGCGTAGAGGTCCACTT
GAPDH fw	CTTTGGTATCGTGGAAGGACT
GAPDH rv	CCAGTGAGCTTCCCGTTCAG
hRIG-I (DDX58) fw	TCCTTTATGAGTATGTGGGCA
hRIG-I (DDX58) rv	CCAGCATTACTAGTCAGAAGG
IFNb fw	GTCTCCTCCAAATTGCTCTC
IFNb rv	ACAGGAGCTTCTGACACTGA
IL8 fw	ACTGAGAGTGATTGAGAGTGGAC
IL8 rv	AACCCTCTGCACCCAGTTTTC
MDA-5 (IFIH1) fw	GCCCGCTACATGAACCCTG
MDA-5 (IFIH1) rv	CAGCAATCCGGTTTCTGTCTT
RNaseL fw	GTAAACGCCTGTGACAATATGGG
RNaseL rv	AGATGCGTAATAGCCTCCACAT
RP49 fw	GACGCTTCAAGGGACAGTATCT
RP49 rv	AAACGCGGTTCTGCATGAG
SINV fw	CCACTACGCAAGCAGAGACG
SINV rv	AGTGCCCAGGGCCTGTGTCCG
SINV fw (fly)	CAAATGTGCCACAGATACCG
SINV rv (fly)	ATACCCTGCCCTTTCAACAA
TotM fw	GCTGGGAAAGGTAAATGCTG
TotM rv	AGGCGCTGTTTTTCTGTGAC
Vago fw	TGCAACTCTGGGAGGATAGC-
Vago rv	AATTGCCCTGCGTCAGTTT
vir-1 fw	GATCCCAATTTTCCCATCAA
vir-1 rv	GATTACAGCTGGGTGCACAA

CLONING

myc-dAgo2 fw
CGGTCTAGAATGGAACAAAAACTTATTTCTGAAGAAGATCTCGGAAAAAAAGATAAGAA
C
myc-dAgo2 rv
CCGGTCGACTTATCACTATCAGACAAAGTACATGGG
V5-R2D2 fw
CGCGCTAGCATGGGTAAGCCTATCCCTAACCCTCTCCTCGGTCTCGATTCTACGGATAACA
AGTCAGCCGTA
V5-R2D2 rv
CGCCTCGAGTTATCACTATTAAATCAACATGGTGCG
Dicer-2 cDNA fw
GGGGACAAGTTTGTACAAAAAAGCAGGCTTCGAAGATGTGGAAATCAAGCCTCGCG
Dicer-2 cDNA rv
GGGGACCACTTTGTACAAGAAAGCTGGGTCTTATCACTATTAGGCGTCGCATTTGCTTAGC
TGC

A

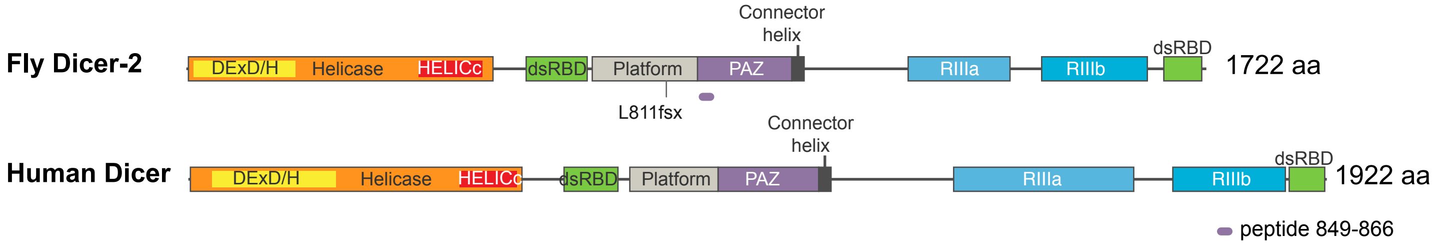

B

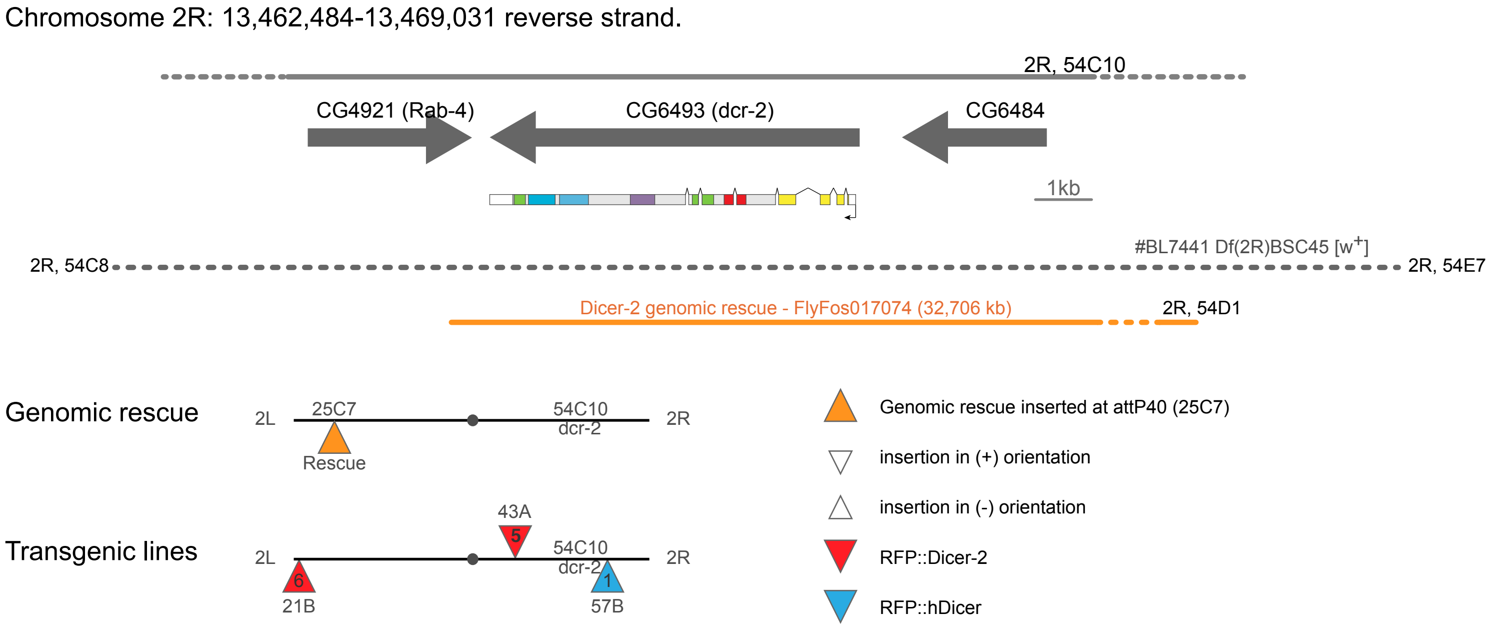

Girardi et al. Fig S1

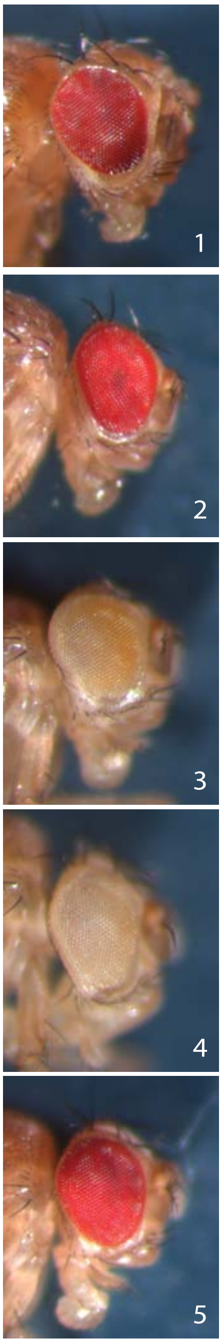

Girardi et al. Fig S2

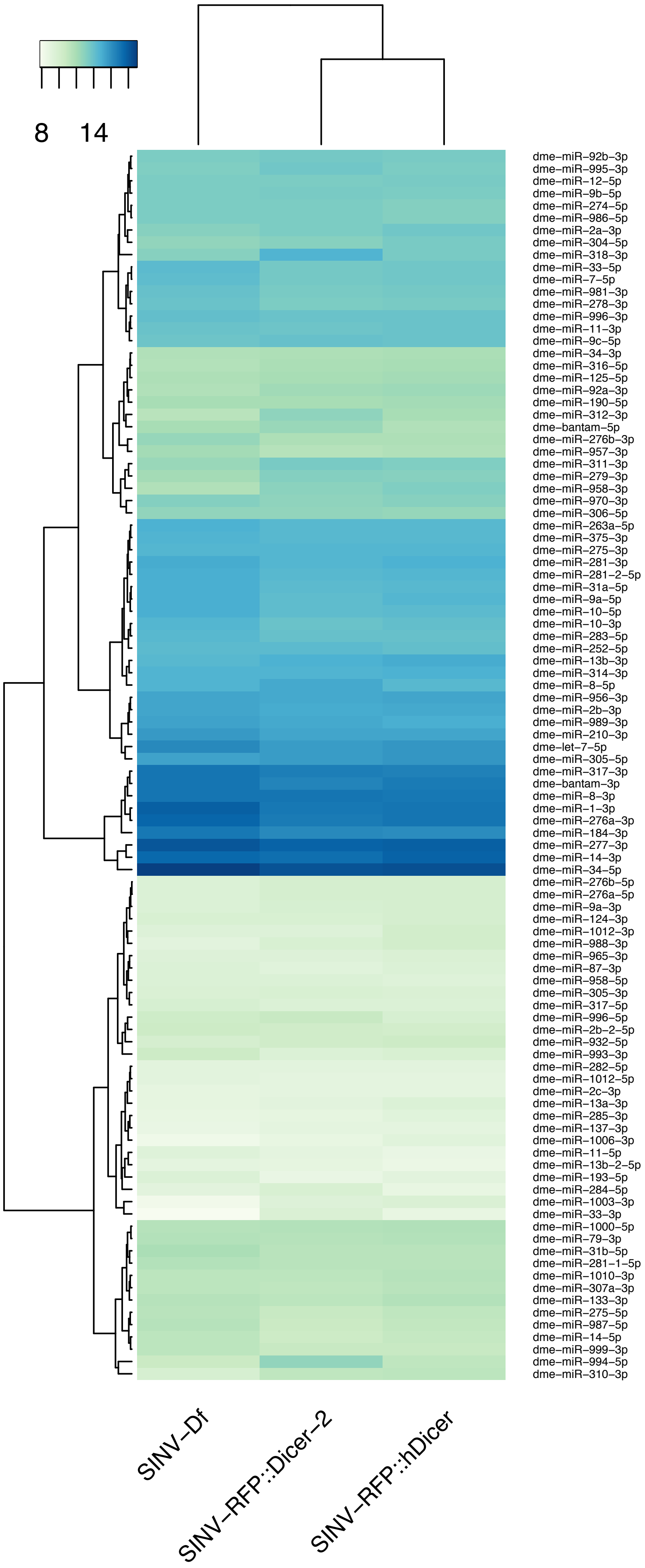

Girardi et al. Fig S3

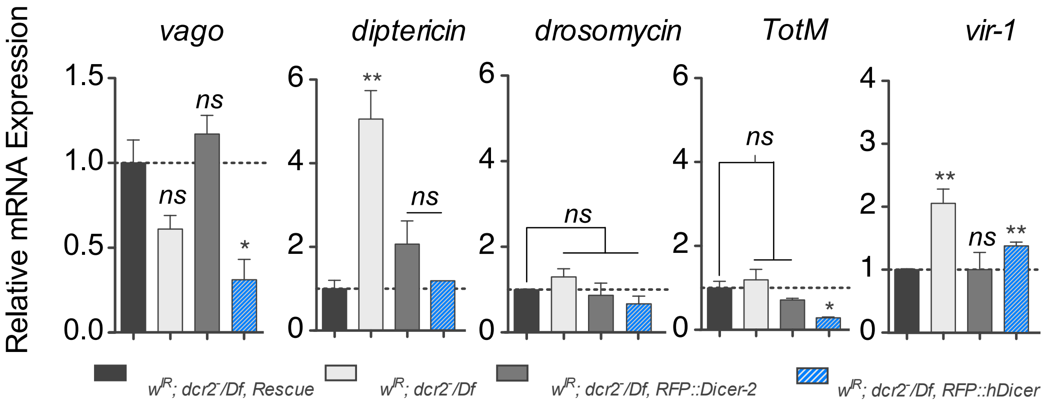

Girardi et al. Fig S4

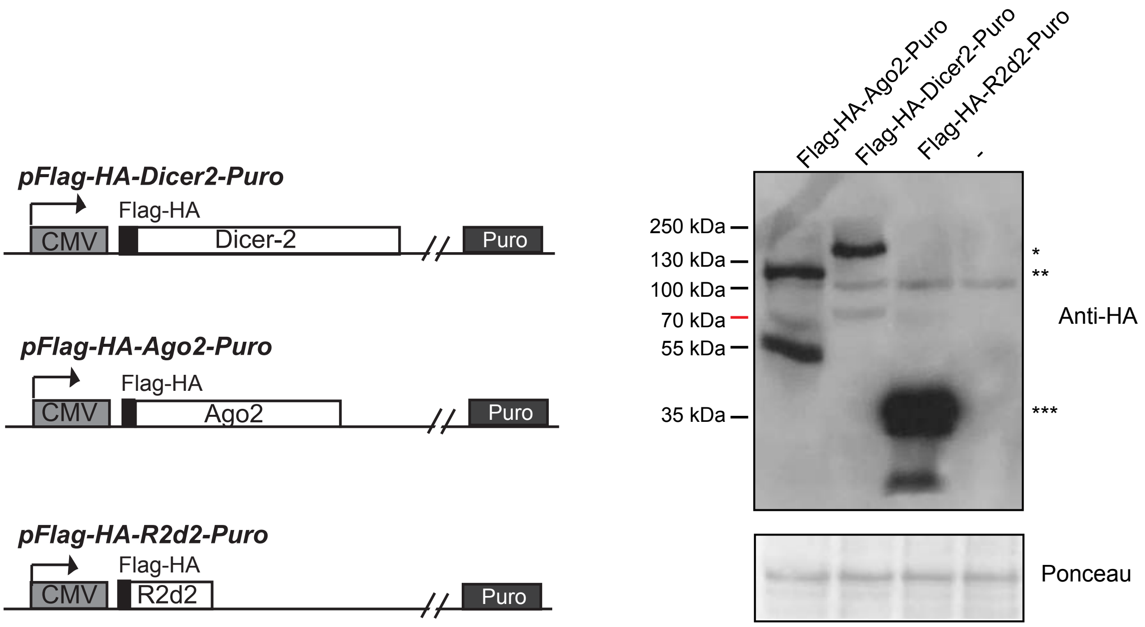

Girardi et al. Fig S5

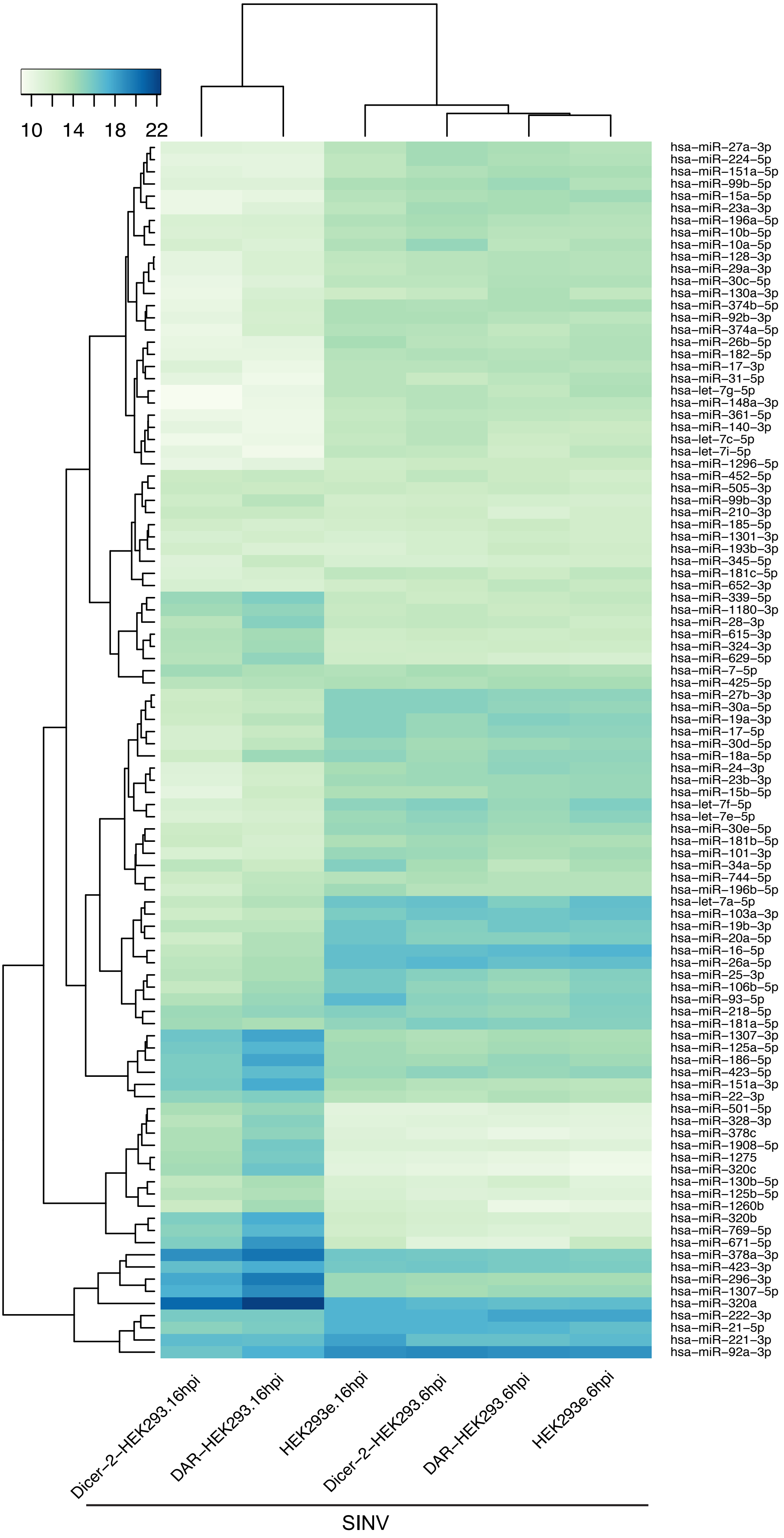

Girardi et al. Fig S6

DAR-HEK293 16 hpi

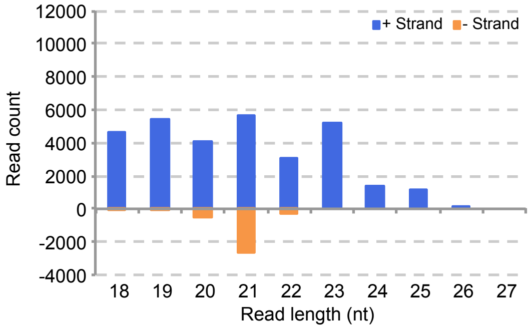

Dicer-2-HEK293 16 hpi

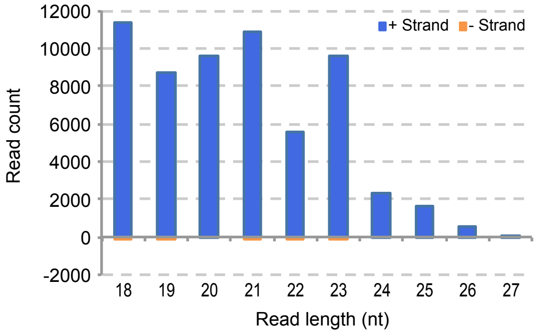

HEK293e 16hpi

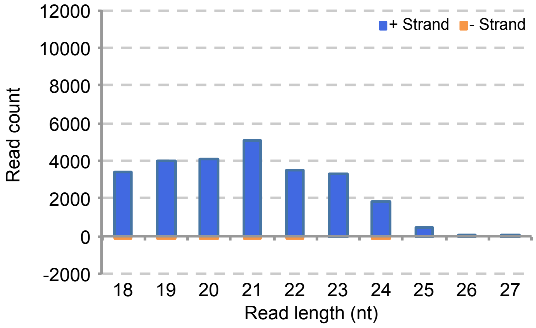

Girardi et al. Fig S7

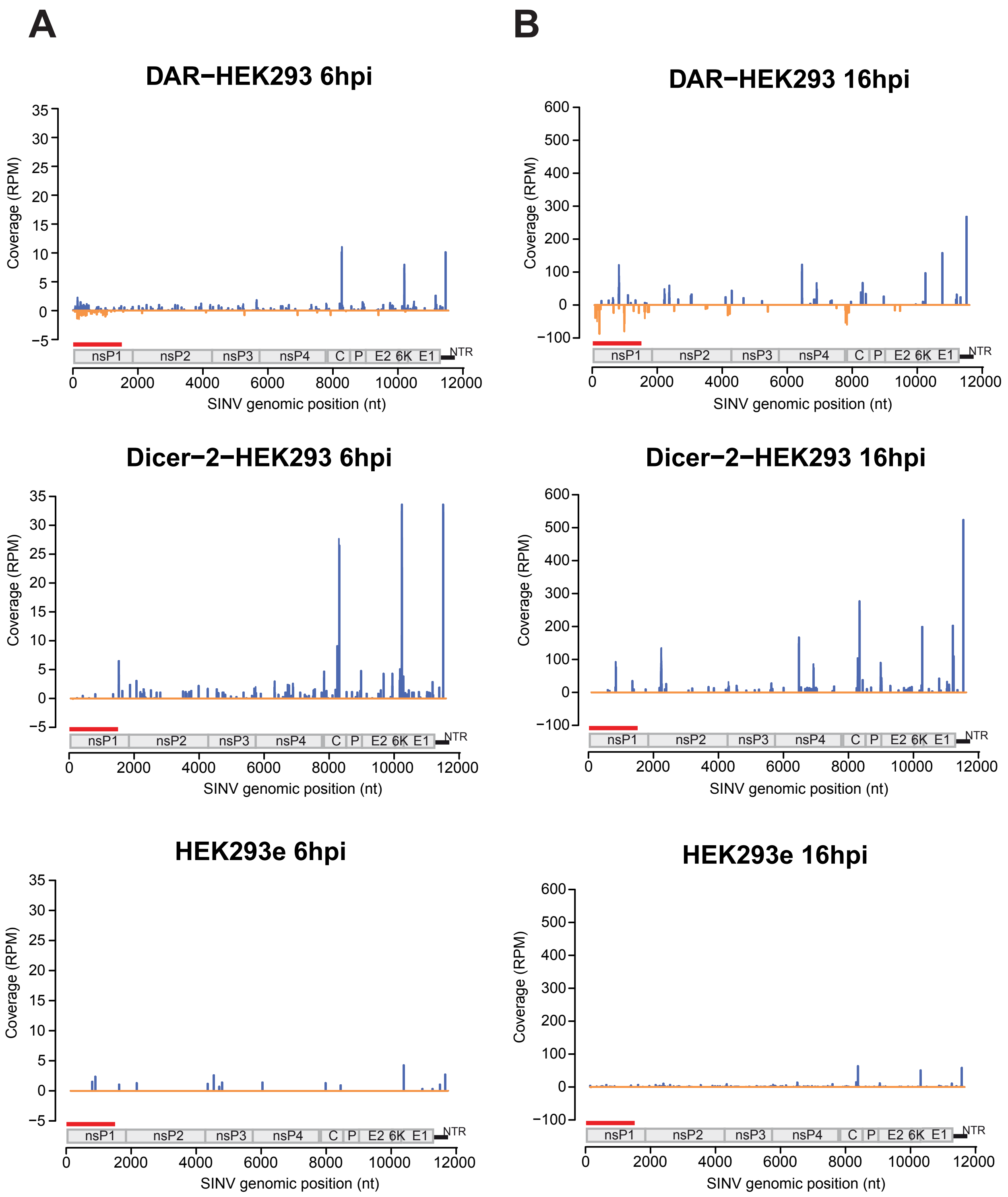

Girardi et al. Fig S8

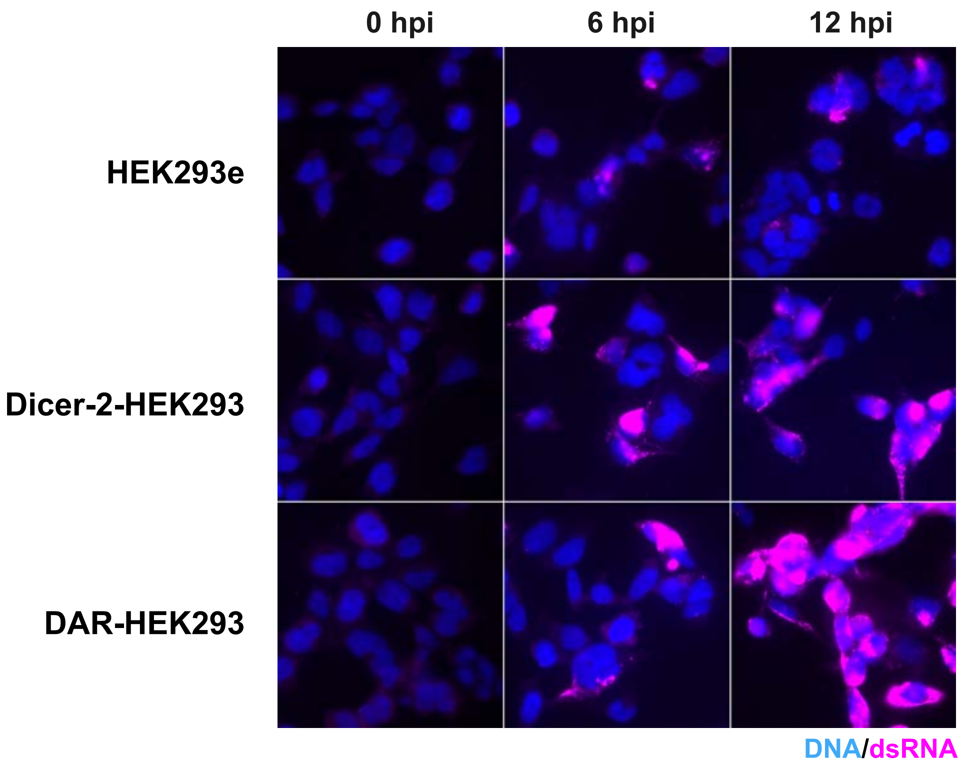

Girardi et al. Fig S9

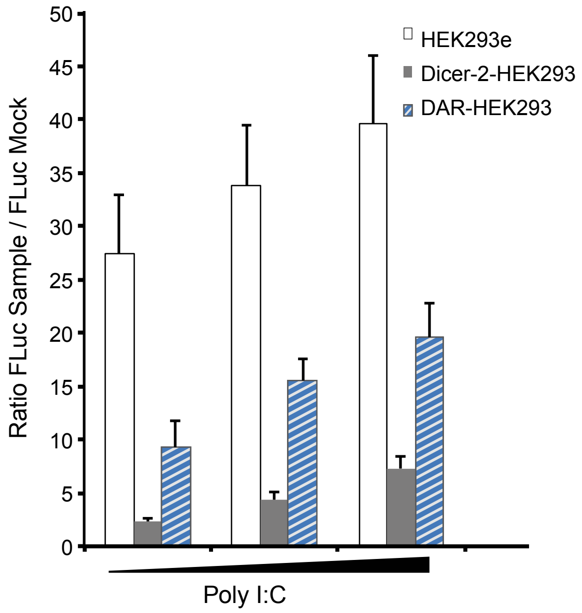

Girardi et al. Fig S10
